# Experimental Investigation on the Extraction and Characterization of Waste Plastic Pyrolysis Oil (WPPO) Obtained From Polypropylene Plastic Waste

**DOI:** 10.1155/tswj/6317016

**Published:** 2024-12-23

**Authors:** Abdulbasit I. Patel, Sumit Das Lala, Imran Molvi, Payel Deb, Abhijit Bhowmik

**Affiliations:** ^1^Department of Mechanical Engineering, Parul Institute of Engineering and Technology, Parul University, Waghodia, Vadodara 391760, Gujarat, India; ^2^Department of Mechanical Engineering, Parul Institute of Engineering and Technology, Parul University, Waghodia, Vadodara 391760, Gujarat, India; ^3^Mechanical Engineering, Parul Institute of Engineering and Technology, Parul University, Waghodia, Vadodara, 391760, Gujarat, India; ^4^Department of Mechanical Engineering, Dream Institute of Technology, Kolkata, 700104, India; ^5^Centre for Research Impact & Outcome, Chitkara University Institute of Engineering and Technology, Chitkara University, Rajpura, 140401, Punjab, India

**Keywords:** landfills, plastic incineration, plastic waste, plastic waste management strategies, pyrolysis, recycling

## Abstract

Plastics are basically long-chain hydrocarbon compound synthesizes from nonrenewable liquid petroleum products. Since plastics have special and variety of features such as easy availability and handling, light weight, energy efficiency, nondegradable nature, cheap, faster production, and design flexibility, it has gained wide popularity in short time period and has become indispensable part of day-to-day life. The increasing usage and production of plastic with exponential rate have resulted in increasing plastic waste disposal problems which may cause adverse effect on environment and human health. Moreover, fast exhaustion of nonrenewable fossil fuel has also become a major problem. To encounter both the problem at a same time, plastic waste conversion method has come into picture. Several plastic waste conversion methods such as landfills, plastic incineration, and recycling are available out of which recycling has gained a lot of interest. One of the important recycling methods is pyrolysis, which is referred as most suitable method due to its advantages such as flexible, easy in handling, less intense sorting, less labor intensive, and high-quality liquid oil extraction. The gaseous by-product also has high calorific value. In the present study, an attempt has been made to produce alternative fuel from waste polypropylene plastic. The study further aims to compare the properties of the obtained WPPO with diesel and blend of WPPO and diesel to ascertain its feasibility for engine runs.

## 1. Introduction

Plastics are basically long chain of polymer composed of hydrogen and carbon which is generally synthesized from petroleum-based products. Due to its variety of features such as nondegradable nature, cheap, easily available and handling, light weight, energy efficiency, faster production, and design flexibility, it has gained wide popularity in short time period [1]. Having this kind of applications, it is obvious that plastic has now become indispensable parts of our daily life and household [2]. The increasing demand of plastic products also increases the accumulation of plastic waste that endangers the environment because of their disposal problems. The rising plastics demand also led to the exhaustion of nonrenewable crude oil as plastics are petroleum-based material. More than 100 million tons of plastics are produced every year worldwide, and the waste plastic is discarded in overflowing plastic bins and landfills [3]. As per data received from Association of Plastic Manufacturers Europe (APME), the global production of plastics has more than 280 million tons in 2011, and it is increasing with exponential rate. Further the data from global plastic production, the worldwide production of plastics reached a staggering 400.3 million metric tons in 2022 as published by Statista Research Department, June 28, 2024. In 1990, the global production capacity was estimated by 80 million tons, and production rate was calculated approximately 5% per year [4]. In the last few decades, consumption and hence production of plastic have increased. From 1950–2015, 8300 million tons of plastics are produced globally, and from this, 6300 million tons of waste are generated. Out of this, only 9% waste is recycled, and 12% incinerated, whereas 79% are thrown in landfills [5]. To achieve the modern world's demand, more than 1.3 billion metric tons of plastic are manufactured each year. With developing human population, the utilization and production of plastic is increasing at an alarming rate [6]. Growing demand of plastic material also increases the growth of plastic waste that is threat to the environment because of their disposal issue [7]. Waste plastics are nonbiodegradable that may undergo photodegradation which can further results it into plastic dust formation that may get in to food chain causing serious health issues [8]. Although plastic become inseparable part of today's world due to its special and wide range of features, simultaneously waste plastics have created very big environmental challenges due to bulk quantities and disposal problem [9]. Mostly, large proportion of plastic waste is makeup by thermoplastics, and its quantity is growing continuously worldwide. Disposal of this plastic waste has become big threat to environment because of their huge amount and nondegradable nature for long time period. Plastics are derived from petroleum products having primarily hydrocarbons but also can contain additives such as colorants, antioxidants and stabilizers. The destruction by incineration of which gives air pollution because of releasing of harmful smokes such as dioxins and hydrogen chloride, air borne particles and carbon dioxide. Recycling of plastic is one of the best options [2]. Moreover, on another side, plastics are synthesized from petroleum products and increased the production of plastic material which also causes quick exhaustion of nonrenewable petroleum products [10].

Diesel engines are most efficient and widely used as prime movers, and for long-term energy protection, it is needed to develop alternative fuels which is as compatible as conventional petroleum-based fuels. Specially, in India, demand for diesel is roughly six times more than gasoline. So, finding alternatives to diesel is a natural choice [11]. Conversion of plastic waste into fuel that is “waste to energy” has two advantages. First, it recycles plastic waste which reduces plastic waste pollution problem, and secondly, some amount of energy in terms of fuel can be obtained from waste [12].

Plastics are most common material in day-to-day life which may be classified as per their chemical structure and branches, process of production, density, applications, and other properties. However, to create ease in recycling process, Society of Plastic Industry (SPI) has defined code system termed as “Resin Identification Code System” which classifies plastics into seven groups according to their chemical structure and applications [13]. These seven types of plastics are marked as follows on different kind of plastics products: (1) polyethylene terephthalate (PET), (2) high-density polyethylene (HDPE), (3) polyvinyl chloride (PVC), (4) low-density polyethylene (LDPE), (5) polypropylene (PP), (6) polystyrene (PS), and (7) other. Government municipalities, environmental authorities, social communities, and different other local communities have developed different norms and environmental safety legislation rules so that we can minimize adverse effect of plastic waste pollution [14]. To minimize the plastic waste disposal problem, various waste management strategies developed and are in use such as landfills, incineration, and recycling. However, the reduction of plastic waste through landfills makes it mandatory to take many precautions to avoid further side effects such as groundwater contamination and land pollution [15]. Around 10% of household waste is plastic waste and is generally thrown on the landfills [16]. Incineration is one of the alternatives to landfills. In this method, waste is burn in the oxygen. Chemically, it is referred as complete combustion that dispenses water molecules and carbon dioxide in the atmosphere [17]. Residues after incineration process are mixture of volatile chemicals, ash, and some amount of hydrochloric acid (HCl). These residues may contain harmful gases as well as hazardous chemicals. On the other hand, all types of plastic waste are not good at combustion perfectly, some resists oxygen heating, and some are explosive. So, precaution must be needed while incineration of waste to avoid accidents [18, 19]. Recycling prefers over landfilling and incineration due to disadvantages of these two methods which are serious concern for society as well environment as it pollutes land and environment, as well threat to ground water contamination and public health [20]. Recycling decreases pollution from ecosystem, requires less amount of energy, and helps in conservation of nature. It reduces fast occupying landfills and helps in the fossil fuel consumption demand. In addition, it also helps in sustainable lifestyle and contributes in economy [21]. Recycling of plastic waste is energy intensive, and on another side, there is alarming exhaustion of nonrenewable energy resources especially petroleum-based fuel. Hence, “energy recovery” or “waste to energy” is best option to encounter both problems. To convert waste plastic into fuel, “pyrolysis” is suitable option. It is one of the best techniques for mass to energy conversion with liquid and gaseous products having high calorific values [22]. Pyrolysis is defined as thermal cracking of long-chain polymer molecules into smaller and less complex molecules. Pyrolysis is done in the absence of oxygen at increased temperature and pressure for short time duration. Pyrolysis is proposed by almost all researchers because it can produce liquid oil up to 80 wt % when heated around 500°C temperature [23]. Janarthanan and Sivanandi [24] explored thermal cracking method which extract fuel from waste plastics. Thermal cracking was done with the help of pyrolysis process performed at wide range of temperature. Liquid yield of about 78% (w/w) was obtained between temperature range 370°C–380°C. Plastic fuel extracted was found having similar properties with diesel. Research explored that diesel engine can be operated with plastic fuel at full load. Brake thermal efficiency improved by 6%, as well unburned hydrocarbon reduced by 4% and 2% in CO emission although NOx emission was increased significantly. Results state that plastic fuel is viable solution as replacement to diesel. A review by Vijayakumar and Sebastian [10] provides a concise summary of different types of plastics and the potential of pyrolysis for fuel production. Based on the studies, pyrolysis process is approved as a potential method to generate energy from plastic waste. It resulted in the production of valuable liquid oil, gaseous fuel, and char instead of waste accumulation in landfills and hence contributed heavily to environmental pollution. Çepeliogullar and Pütün [25] conducted pyrolysis on PET at 500°C using fixed-bed reactor. 10°C/min of heating rate was maintained, and nitrogen as sweeping gas was used. Liquid yield obtained was 23.10 wt %, and gaseous yield was approximately 76.90 wt%. Ahmad et al. [26] have conducted pyrolysis process for HDPE at temperature between 300°C and 400°C using steel reactor and nitrogen as fluidizing medium. 80.88 wt % of liquid yield achieved at 350°C and 33.05 wt % of largest amount of solid residue obtained at 300°C. The author further conducted pyrolysis on PP at 250°C–400°C temperature. Liquid yield obtained was 69.82 wt % at 300°C. Pyrolysis of HDPE was done by Kumar and Singh [27], using semibatch reactor at increased temperature of 400°C–550°C. Highest liquid yield and gaseous product obtained were 70.08 wt % and 24.75 wt %, respectively, at 550°C. Marcilla, Beltrán, and Navarro [28] conducted pyrolysis on HDPE at 550°C. Liquid yield obtained was 84.7 wt %, and gaseous yield obtained was 16.30 wt %. Results state that, with higher temperature, higher liquid yield can be achieved. Mastral et al. [29] reported an experiment done on HDPE using fluidized bed reactor at 650°C. Liquid oil derived 68.50 wt % and 31.50 wt % of gaseous product was obtained. Result shows that at high temperature above 550°C, secondary cracking of molecules occurs, and due to this, liquid product further converts into gaseous product, hence liquid yield decreased. Miranda et al. [30] conducted experiment on PVC using batch reactor at 220°C–520°C. Heating rate was 10°C/min. Liquid oil obtained was in the range of 0.45 wt % to 12.78 wt % and 58.20 wt % HCl was obtained. Bagri and Williams [31] experimented on LDPE in fixed bed reactor at 500°C. The experiment was performed for 20 min time duration, and fluidized medium used was nitrogen gas. Liquid yield of highest 95 wt % was achieved with small amount of gaseous product and negligible quantity of char. A work reported by Uddin et al. [32] shows the use of LDPE using batch reactor at 400°C for the extraction of plastic oil. The study reveals that 75.60 wt % of liquid was obtained. Onwudili, Insura, and Williams [33] conducted pyrolysis on LDPE using pressurized batch reactor (0.8–4.3 MPa) at 425°C. Liquid yield 89.50 wt %, gaseous product 10 wt %, and 0.50 wt % of char were obtained. The author further conducted experiment on PS at temperature of 300°C–500°C. Heating rate was 10°C/min, and pressure was in the range of 0.31–1. 6 MPa. Liquid yield was 97 wt % at the temperature of 425°C, and only 2.50 wt % of gas was produced. The outcomes of the literature survey are tabulated in Table 1.

Extensive study of the literature shows that out of the different types of polymers listed above, PP was not properly explored by the researchers. Hence, an attempt is being made in the present study to investigate the potential of PP for extraction of liquid fuel using pyrolysis process.

## 2. Materials and Methods

### 2.1. Feed Material

**Figure 1:**
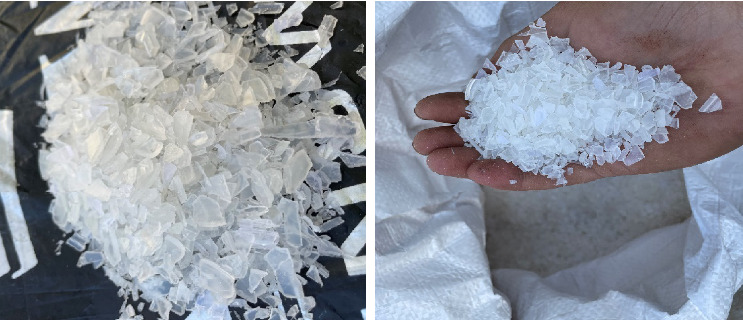
Polypropylene (PP) waste.

PP waste is selected as feedstock raw material, which having high calorific value and largest in municipal solid waste, as well, PP gives good liquid yield and plastic oil extracted from PP waste having more similar properties with conventional fuel. PP waste was collected from one of the plastic industries located in Ranoli, Vadodara. The obtained material is already in shredded and pure form because it is waste generated from large sheets and cubes from plastic industry.  Figure 1 shows PP waste which is used as feedstock raw material.

### 2.2. Experimental Setup

**Figure 2:**
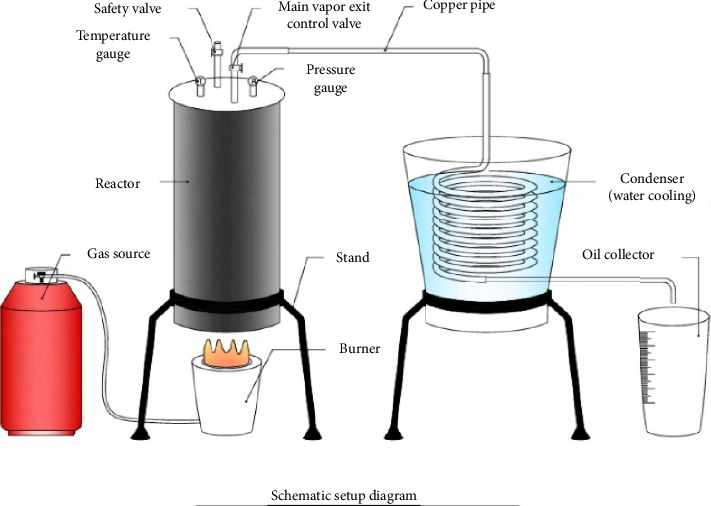
Schematic diagram of pyrolysis setup.

**Figure 3:**
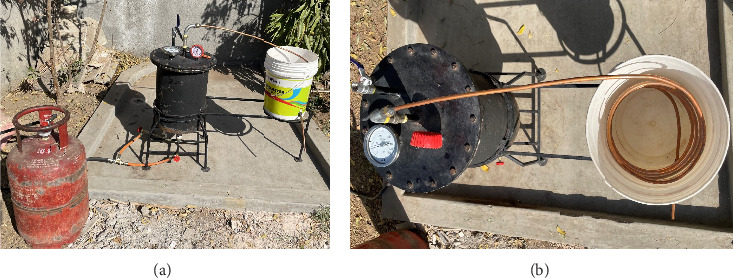
(a) Actual experimental setup for pyrolysis—front view. (b) Actual experimental setup for pyrolysis—top view.

The schematic diagram and actual experimental setup for pyrolysis is shown in Figures 2, 3(a), and 3(b), respectively. It contains main two parts, i.e., reactor and condenser. Reactor is made up of mild steel. Reactor side wall is made up of 8 mm thick plate, and top and bottom plate is 10 mm thick to withstand high temperature and pressure. Reactor is batch type. 15 nut-bolt and head gasket is used to avoid leakage and for perfect fitting of head. On the top of reactor, one main outlet valve from which generated vapor of PP comes out is provided. Valve is used so that the samples can be collected at desired temperature and pressure can develop if desired. Temperature gauge and pressure gauge are mounted on the top of reactor to measure temperature and pressure inside the reactor. Safety valve is also provided which can release unwanted water vapor and extra gases developed at initial phase and also can release pressure in an emergency case. Reactor is heated by gas-burner assembly. Commercial LPG cylinder is used for gas source, and full flame industrial burner is used. Burner is fixed in stand while reactor and condenser are placed on it. Heated PP vapor coming from main outlet valve is passed through copper pipe, which is sinking in condenser. Condenser is made up of copper pipe coil which is sinked in cold water. Condensed liquid oil, i.e., waste plastic pyrolysis oil (WPPO) is collected in oil collector (any bottle, can, etc.), and the gas is released in air.

### 2.3. Experimental Procedure

The trial run was initially started with 3 kg of feed material. Also, the rate of temperature rise is very slow, and the peak temperature achieved was 300°C. Approx. ∼1.5 L of WPPO obtained after 5–6 h of processing. The maximum temperature was achieved with fully opened burner for the entire experimentation. Thereafter, a fall in temperature was noticed which may be attributed to the exhaustion of whole feed material.

**Figure 4:**
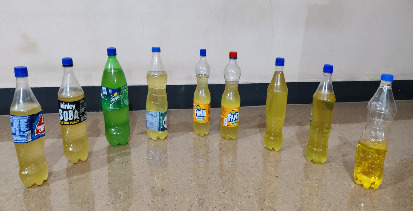
Net WPPO collected through pyrolysis of 6 kg PP waste.

In regard to the first trial, for the second trial, it was observed that more quantity of feed material can be incorporated. Hence, 6 kg of PP plastic waste was fed in batch type reactor to obtain require quantity of WPPO. Initially, safety valve is opened for a while, to release the water vapor and extra gases. After achieving 150°C, main exit valve is opened. It took around 30–40 min to reach 150°C. Generated vapor is passed through condenser. Both the valves were opened during the operation, and safety valve was opened for a while to release the water molecules and extra gases. Thereafter, condensed WPPO collected in oil collector. Sample collection started after 150°C was achieved by burner. Approximately, 2 h and 40 min time was required to reach 370°C from 150°C. Peak temperature achieved was 370°C. The entire process of pyrolysis was of approx. 3.5 h, and the WPPO obtained was around 6 L which is approx. 4.8 kg as shown in Figure 4.

### 2.4. Characterization Techniques

The obtained WPPO was then characterized for different biofuel properties. The properties include density (gm/cc), specific gravity (unit), API gravity (° API), kinematic viscosity (cSt at 40°C), flash point (°C), fire point (°C), cloud point (°C), pour point (°C), and calorific value (kCal/kg).

1. Density: For a liquid, density is the mass contained in a unit volume. More specific name for density is volumetric mass density.
2. Specific gravity: Specific gravity (also referred to as relative density) is the ratio of the density of a liquid compared to the density of water. It is the unitless quantity.
3. API gravity: API gravity is extensively used as a measure of the grade of crude oil products. It is a measure of the heaviness of crude oil products when compared with water. The API gravity is a function of the relative density or specific gravity of the crude oil liquid. The formula for API gravity is [API = (141.5/specific gravity)–131.5]. The API gravity parameter is dimensionless, based on the equation. However, it is often used with a degree symbol, such as the degree of API gravity (°API).
4. Kinematic viscosity: Kinematic viscosity is a measure of a fluid's internal resistance to flow under gravitational forces. It is determined by measuring the time in seconds, required for a fixed volume of fluid to flow to a known distance by gravity through a capillary within a calibrated viscometer at a closely controlled temperature. This value is converted to standard units such as centistokes (cSt) or square millimeters per second. Viscosity reporting is only valid when the temperature at which the test was conducted also is reported.
5. Calorific value: It is the amount of heat produced by unit volume of a substance on complete combustion. It is expressed in kilojoules/kilogram.
6. Flash point: It is the lowest temperature at which the oil gives off vapor that will ignite where a flame is passed over surface of the oil.
7. Fire point: It is the lowest temperature at which sample ignites and continues to burn for at least 5 s. The flash and fire points can be used to determine the transportation and storage temperature requirements for oils.
8. Cloud point: The cloud point of a fluid is the temperature at which dissolved solids are no longer completely soluble, precipitating as a second phase giving the fluid a cloudy appearance.
9. Pour point: Pour point is used as an indication of the lowest temperature at which a fuel can be pumped. Cloud point and pour point are the important cold flow properties of petroleum fuels. It determines the functional efficiency of fuels at lower temperatures.

## 3. Results and Discussion

The obtained WPPO was then tested for different biofuel properties. A blend of the biofuel was being made using WPPO (20%) + diesel (80%). The test was repeated thrice, and the average of the results was considered. The test results were further compared with the properties of diesel as well the WPPO blend, and the comparison results are listed in Table 2.

The observations of different properties from Table 2 and their probable impact on the biofuel properties are discussed as follows:

1. Density: Density is an important property of a fuel oil. If the density of fuel is high, then the fuel consumption will be less. On the other hand, the oil with low density will consume more fuel which may cause damage to the engine. Therefore, too low or too high density of fuel oil is not desirable [34]. From the result table, it is observed that derived pure WPPO density is slightly lower than diesel, whereas density of blend (WPPO and diesel) is almost nearer to the pure diesel.
2. API gravity: It is generally used to measure the grade of crude oil products. API gravity of WPPO is calculated as 55.35 API while for diesel, it is 41.6 API. So, WPPO is fall under extra light category. But blend and pure diesel both are under light category.
3. Viscosity: Viscosity is another very essential parameter to guarantee a good atomization property of a fuel [35]. If the kinematic viscosity value is low, it will influence the oil fuel quality, giving it a low heating value [36]. The higher the viscosity, the higher the fuel consumption, engine temperature, and load on the engine. On the other hand, if the viscosity of oil is too high, excessive friction may take place. Measured viscosity of blend is slightly higher than diesel.
4. Calorific Value: Calorific value is an important property of diesel fuels because it determines the energy content in the fuel. A higher calorific value indicates that a larger amount of energy is produced. Calorific value of pure WPPO found as 8700 kCal/kg, but it reaches upto 9600 kCal/kg when it is blend with 80% diesel which is nearer to pure diesel.
5. Flashpoint: It is a very important property for the storage and handling of fuels. Flash point is used to characterize the fire hazards of fuels. Flashpoint is a good indicator of diesel fuel contamination with some more volatile chemicals [35]. The fire point is used to assess the risk of the materials ability to support combustion. Generally, the fire point of any liquid oil is considered to be about (5–10) °C higher than the flash point. Measured flash and fire point value for WPPO and blend both are closer to the pure diesel.
6. Cloud and pour point: Cloud and pour point are the important cold flow properties of petroleum fuels. It determines the functional efficiency of fuels at lower temperature. From the result, it is observed that WPPO and blend are having closer value with diesel.

**Figure 5:**
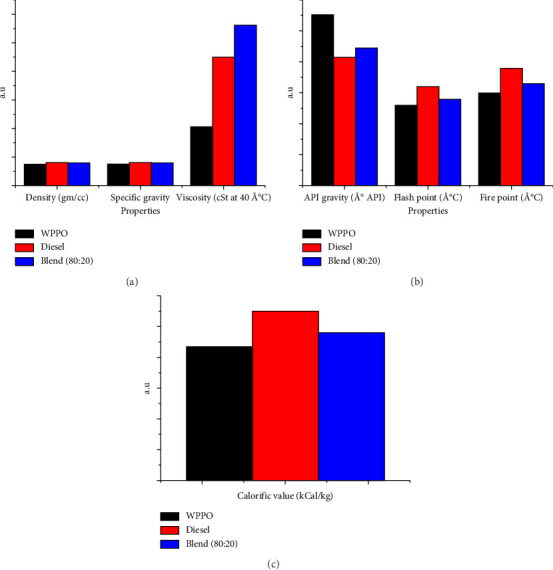
(a)–(c) The variation of properties for the WPPO, diesel, and blend composition.

Figure 5(a), 5(b), and 5(c) show the variation of different properties for the WPPO, diesel, and blend composition.

The results of all the properties for the blended samples are comparable to the properties of diesel. Hence, the properties obtained for the blended samples show that it can be effectively utilized for the engine test runs.

## 4. Conclusions

The present study focuses on the extraction of plastic oil from PP plastic waste using pyrolysis method. There are mainly seven types of plastic waste found, i.e., PET, HDPE, PVC, LDPE, PP, PS, and other. However, PP plastic is not being explored by the researchers properly. Hence, in the present study, an attempt has been made to synthesize WPPO from PP plastic waste. The characterization of the obtained oil was carried out, and their properties were compared to both diesel and the blend of WPPO-diesel. From the experimental study and property comparison, the following conclusions are drawn:

• Pyrolysis is suitable and easy process for waste to energy that is converting plastic waste into WPPO fuel.
• Pyrolysis of PP gives very good quality of WPPO and high liquid yield.
• From comparing properties of WPPO with diesel, it was noted that PP-WPPO blend is of very good quality having comparable fuel properties. Moreover, it was feasible to be blended with diesel.
• From comparing WPPO-blend properties with diesel, it was observed that PP-WPPO blend is having very good quality as compared with diesel.

## Figures and Tables

**Table 1:** Key highlights from literature.

Sl no	Plastic type	Methodology used	Liquid yield	Temperature range
1	Polypropylene, polyethylene, and polystyrene	Pyrolysis	78 wt.%	370°C–380°C
2	PET	Pyrolysis	23.10 wt %	500°C
3	HDPE	Pyrolysis	80.88wt %	300°C–400°C
4	PP	Pyrolysis	69.82 wt %	250°C–400°C
5	HDPE	Pyrolysis	70.08 wt %	400°–550°C
6	HDPE	Pyrolysis	84.7 wt %	550°C
7	HDPE	Fluidized bed reactor	68.50 wt %	650°C
8	PVC	Batch reactor	0.45 wt % to 12.78 wt %	220°C–520°C
9	LDPE	Fixed bed reactor	95 wt %	500°C
10	LDPE	Batch reactor	75.60 wt %	400°C
11	LDPE	Pressurized batch reactor	89.50 wt %	425°C
12	PS	Pyrolysis	97 wt %	300°C–500°C

**Table 2:** Properties of WPPO, diesel, and blend.

Sr. no.	Property	WPPO	Diesel	WPPO (20%) + diesel (80%)
1	Density (gm/cc)	0.7545	0.8150	0.8010
2	Specific gravity	0.757	0.8175	0.8034
3	API gravity (° API)	55.35	41.6	44.62
4	Viscosity (cSt at 40°C)	2.067	4.510	5.617
5	Flash point (°C)	26	32	28
6	Fire point (°C)	30	38	33
7	Cloud point (°C)	−6 to −9	−9 to −12	−7 to −11
8	Pour point (°C)	−18 to −21	−15 to −19	−19 to −23
9	Calorific value (kCal/kg)	∼8700	∼10,500–11,000	∼9600

## Data Availability

The data will be available on request.
